# Sustainable Approach for Peroxygenase-Catalyzed Oxidation Reactions Using Hydrogen Peroxide Generated from Spent Coffee Grounds and Tea Leaf Residues

**DOI:** 10.1021/acsomega.2c02186

**Published:** 2022-06-01

**Authors:** Hideaki Kawana, Toru Miwa, Yuki Honda, Toshiki Furuya

**Affiliations:** † Faculty of Science and Technology, Tokyo University of Science, 2641 Yamazaki, Noda 278-8510, Chiba, Japan; ‡ Department of Chemistry, Biology, and Environmental Science, Faculty of Science, Nara Women’s University, Kitauoyanishi-machi, Nara 630-8506, Japan

## Abstract

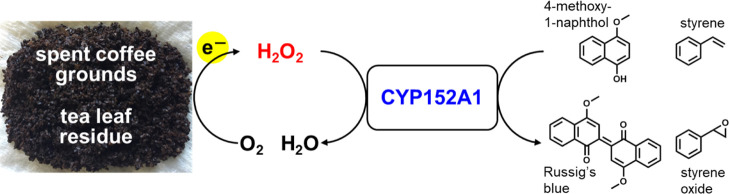

Peroxygenases are promising catalysts for use in the oxidation of chemicals as they catalyze the direct oxidation of a variety of compounds under ambient conditions using hydrogen peroxide (H_2_O_2_) as an oxidant. Although the use of peroxygenases provides a simple method for oxidation of chemicals, the anthraquinone process currently used to produce H_2_O_2_ requires significant energy input and generates considerable waste, which negatively affects process sustainability and production costs. Thus, generating H_2_O_2_ for peroxygenases on site using an environmentally benign method would be advantageous. Here, we utilized spent coffee grounds (SCGs) and tea leaf residues (TLRs) for the production of H_2_O_2_. These waste biomass products reacted with molecular oxygen and effectively generated H_2_O_2_ in sodium phosphate buffer. The resulting H_2_O_2_ was utilized by the bacterial P450 peroxygenase, CYP152A1. Both SCG-derived and TLR-derived H_2_O_2_ promoted the CYP152A1-catalyzed oxidation of 4-methoxy-1-naphthol to Russig’s blue as a model reaction. In addition, when CYP152A1 was incubated with styrene, the SCG and TLR solutions enabled the synthesis of styrene oxide and phenylacetaldehyde. This new approach using waste biomass provides a simple, cost-effective, and sustainable oxidation method that should be readily applicable to other peroxygenases for the synthesis of a variety of valuable chemicals.

## Introduction

P450 monooxygenases are a superfamily of heme-containing proteins that introduce one oxygen atom derived from molecular oxygen (O_2_) into an organic molecule. P450 monooxygenases catalyze the direct oxidation of a variety of compounds in a regio- and stereo-selective manner under ambient conditions.^1−4^ Thus, P450 monooxygenases are promising catalysts for the oxyfunctionalization of chemicals.^5−8^ A heme moiety in the catalytic center of P450 activates O_2_ using electrons transferred from NAD(P)H by reductase components. The resulting active oxidant, known as compound I, oxidizes substrate molecules. However, because P450 monooxygenases require NAD(P)H as a coenzyme, this biotechnological process can be complicated as a continuous supply of this expensive coenzyme is required for P450s to achieve high-yield production of oxidized chemicals.^9,10^ In contrast, P450 peroxygenases utilize hydrogen peroxide (H_2_O_2_) instead of O_2_ to generate compound I. Because P450 peroxygenases do not require NAD(P)H, these enzymes are advantageous for practical applications.^11,12^ CYP152A1 of *Bacillus subtilis* is a prototypical bacterial P450 peroxygenase that catalyzes the hydroxylation of long-chain saturated fatty acids.^13,14^ Intriguingly, the substrate specificity of CYP152A1 can be altered by decoy molecules such as short-chain fatty acids.^15^ For example, in the presence of short-chain fatty acids, CYP152A1 catalyzes the epoxidation of the non-natural substrate styrene.^16^ In addition to CYP152A1, a variety of other P450 peroxygenases reportedly catalyze direct oxidation reactions in the synthesis of important chemicals.^17,18^

P450 peroxygenases require H_2_O_2_ as an oxidant. Currently, H_2_O_2_ is manufactured via the anthraquinone process,^19−21^ in which anthraquinone is reduced to anthrahydroquinone by hydrogen gas on a palladium catalyst in an organic solvent. The resulting anthrahydroquinone reduces O_2_ to produce H_2_O_2_, which is recovered via liquid–liquid extraction. This multistep method requires significant energy input and generates considerable waste, which negatively affects process sustainability and increases production costs.^19−21^ Thus, generating H_2_O_2_ for peroxygenases on site using an environmentally benign method would be advantageous. Several enzymatic and photocatalytic methods for in situ H_2_O_2_ generation have been reported.^17,18^ For example, glucose oxidase can be used to couple the oxidation of glucose to the reductive activation of O_2_ to form H_2_O_2_, which drives peroxygenase-catalyzed oxidation reactions.^22,23^ TiO_2_-based semiconductors that generate H_2_O_2_ from O_2_ under light irradiation using sacrificial electron donors such as methanol have also been evaluated.^24,25^ These in situ approaches enable the generation of low concentrations of H_2_O_2_, thereby alleviating the problem of inactivation of P450 peroxygenases by the oxidant. However, enzymatic and photocatalytic methods are more expensive than the direct addition of H_2_O_2_ due to the requirement for catalysts (e.g., glucose oxidase and TiO_2_-based semiconductors) and electron donors (e.g., glucose and methanol) to produce H_2_O_2_.

Coffee is the most popular non-alcoholic beverage in the world, with a total of 10 million tons of coffee beans consumed in 2020.^26^ Coffee consumption generates large amounts of spent coffee grounds (SCGs) that are mostly discarded as waste due to lack of economic value. By comparison, tea is the world’s second most popular non-alcoholic beverage,^26^ and the consumption of vast amounts of tea also generates large amounts of tea leaf residues (TLRs) that pose a similar waste problem to that of SCG. Increased awareness in recent years of the need for waste reduction and environmental protection has highlighted the need to find ways to valorize SCG and TLR. Because these waste biomass products consist of a large number of organic compounds such as polysaccharides and polyphenols, they have attracted attention as bioresources for fuels and other valuable chemicals.^27−29^ Polyphenols such as chlorogenic acid and caffeic acid are present in high levels in coffee, and catechins are abundant in tea.^30,31^ These are biologically active molecules that exert a variety of beneficial effects, including antioxidant and anticancer activities.^32,33^ Intriguingly, polyphenols in coffee and tea also act as pro-oxidants, reducing O_2_ to form the oxidant H_2_O_2_ (Figure S1), which is associated with the antimicrobial activity of these compounds.^34−37^ From the viewpoint of industrial applications, we hypothesized that polyphenol-containing SCG and TLR could be used for the production of H_2_O_2_. To date, however, there have been no reports concerning H_2_O_2_ production from these waste biomass sources.

Here, we report a novel SCG- and TLR-driven approach for generating H_2_O_2_ to promote peroxygenase-catalyzed oxidation reactions. We first found that these waste biomass products react with O_2_ and effectively generate H_2_O_2_ in sodium phosphate buffer. This approach provides a simple and cost-effective method for the production of H_2_O_2_. The resulting H_2_O_2_ was utilized by the bacterial P450 peroxygenase, CYP152A1. We demonstrate here that H_2_O_2_ produced from SCG and TLR promotes the synthesis of Russig’s blue and styrene oxide via the activity of CYP152A1.

## Results and Discussion

### H_2_O_2_ Production from Waste Biomass

**Figure 1:**
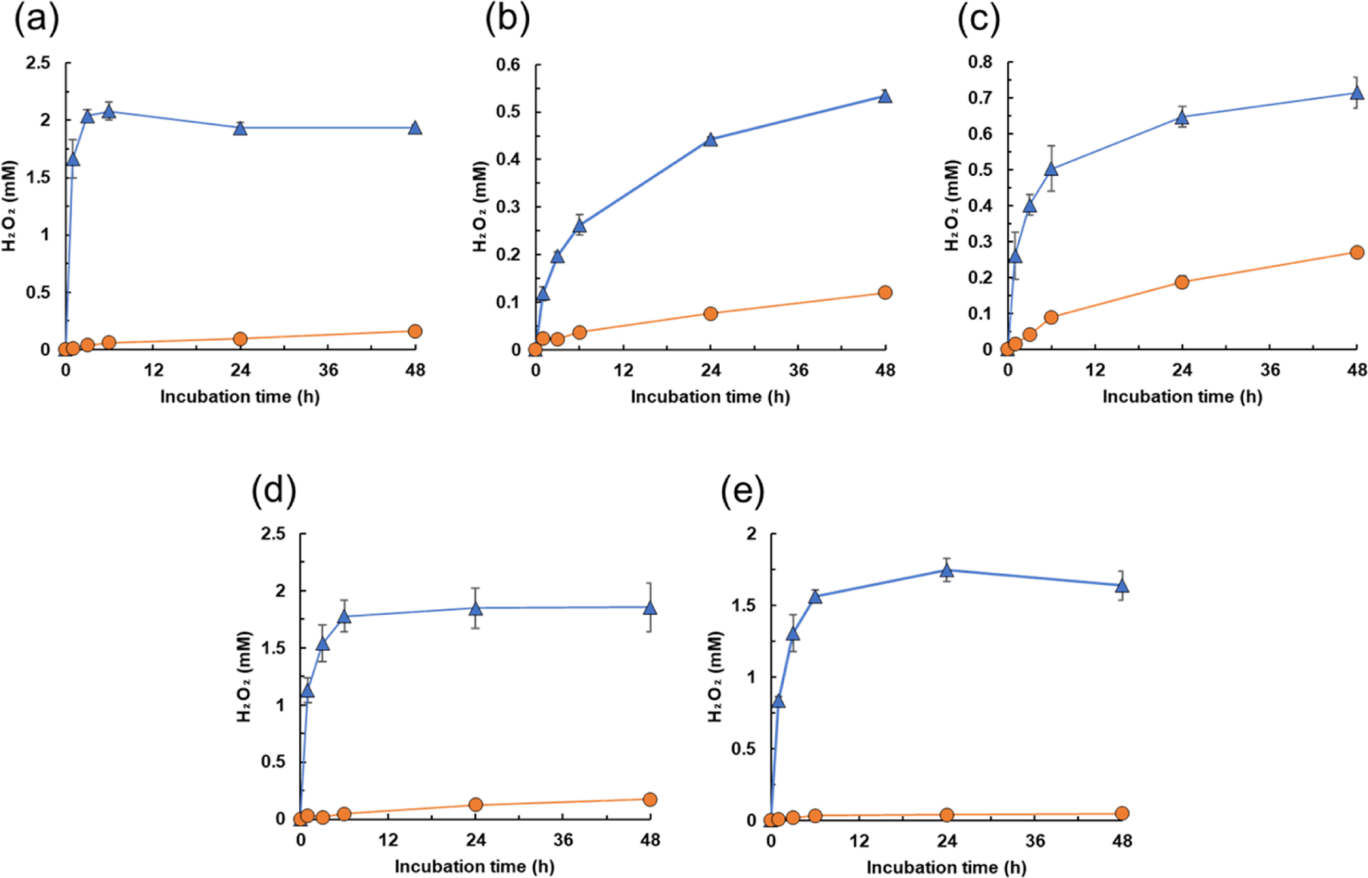
Production of H_2_O_2_ from SCG and TLR. Solutions of pyrogallol (a) coffee (b), SCG (c), tea (d), and TLR (e) were each mixed with water (circles) or sodium phosphate buffer (triangles) and incubated at 30 °C with shaking. Generation of H_2_O_2_ was measured using the FOX assay. Data are the average of three independent experiments, and error bars indicate the standard deviation from the mean.

We first examined the production of H_2_O_2_ from SCG and TLR. After preparation of coffee and tea, the resulting SCG and TLR as waste biomass sources were added to distilled-deionized water. A solution of pyrogallol as a model polyphenol compound was also prepared. The coffee, tea, SCG, TLR, and pyrogallol solutions were each mixed with water or sodium phosphate buffer and incubated at 30 °C with shaking, as described in the Experimental Section. Generation of H_2_O_2_ was measured using a ferrous ion oxidation-xylenol orange assay. Pyrogallol in water produced 0.16 mM H_2_O_2_ in 48 h, as reported previously (Figure 1a).^34^ In addition, we found that SCG and TLR, as well as coffee and tea, yielded H_2_O_2_ (Figure 1b–e). SCG and TLR in water produced 0.27 and 0.05 mM H_2_O_2_, respectively. Furthermore, productivity was strongly enhanced using sodium phosphate buffer (Figure 1). H_2_O_2_ production from SCG and TLR in the buffer continued to increase in a time-dependent manner, reaching 0.72 and 1.64 mM, respectively, by 48 h. Akagawa et al. demonstrated that trace amounts of metal ions (e.g., copper and iron) in sodium phosphate buffer catalyze the reduction of O_2_ by polyphenols to generate H_2_O_2_ (Figure S1).^34^ Considered collectively, these data indicate that SCG and TLR are useful as electron donors for the production of H_2_O_2_.

### Effect of H_2_O_2_ Concentration on CYP152A1-Catalyzed Oxidation

**Scheme 1:**
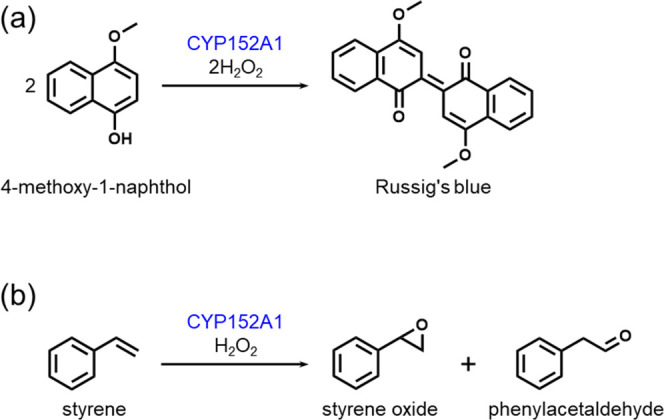
CYP152A1-Catalyzed Oxidation of 4-Methoxy-1-naphthol (a) and Styrene (b)

Before we evaluated CYP152A1-catalyzed oxidation using H_2_O_2_ generated from SCG and TLR, we examined the effect of H_2_O_2_ concentration on the reaction, which has not been previously reported. His-tagged CYP152A1 was produced in *Escherichia coli* and then purified from the soluble fraction of the cells using a nickel column (Figure S2). In the presence of short-chain fatty acids as decoy molecules, CYP152A1 reportedly catalyzes the oxidation of 4-methoxy-1-naphthol to produce Russig’s blue (Scheme 1a).^38^ In the present study, when CYP152A1 (0.25 mg mL^–1^, 5.0 μM) was incubated with 4-methoxy-1-naphthol (1 mM) and H_2_O_2_ (0.1 mM, 0.25 mM, or 0.5 mM) in the presence of heptanoic acid (10 mM) for 120 s as a model reaction, the mixtures turned blue due to the formation of Russig’s blue (Figure S3). In contrast, incubation of 4-methoxy-1-naphthol, H_2_O_2_, and heptanoic acid without addition of CYP152A1 resulted in no color change (Figure S3), indicating that product formation depends on CYP152A1-catalyzed oxidation. As the initial concentration of H_2_O_2_ was increased, CYP152A1 exhibited a higher turnover frequency (TOF) and produced a higher amount of Russig’s blue (Table 1). Estimated product yields based on H_2_O_2_ were approximately 48%, irrespective of the initial H_2_O_2_ concentration (Table 1). The relatively low product yields might be attributable to uncoupling of H_2_O_2_ consumption from CYP152A1-catalyzed oxidation. Indeed, we confirmed that no H_2_O_2_ remained in the reaction mixture after incubation of CYP152A1, H_2_O_2_ (0.5 mM), and heptanoic acid for 120 s, both in the presence and absence of 4-methoxy-1-naphthol (Figure S4a). In contrast, almost no change in the amount of H_2_O_2_ was observed in the reaction mixture lacking CYP152A1 (Figure S4a). A recent report indicated that CYP152A1 exhibits catalase activity that competes with the peroxygenase activity during substrate oxidation.^39^ Overall, we found that the amount of Russig’s blue produced by CYP152A1 depends on the initial H_2_O_2_ concentration.

**Table 1:** Effect of H_2_O_2_ Concentration on CYP152A1-Catalyzed Synthesis of Russig’s Blue

H_2_O_2_ (mM)	Russig’s blue (μM)	product yield (%)^a^	TOF (min^–1^)^b^
0.1	24.0 ± 2.7	48.0 ± 5.4	18.5 ± 3.6
0.25	60.2 ± 1.0	48.2 ± 0.8	41.1 ± 2.1
0.5	120.5 ± 4.0	48.2 ± 1.6	70.4 ± 3.6

a Product yield (%) based on H_2_O_2_ expressed as (2 × Russig’s blue produced [mol])/(H_2_O_2_ added [mol]) × 100.

b TOF (min^–1^) was estimated for the first 10 s of the reaction.

We also examined the effect of H_2_O_2_ concentration on the CYP152A1-catalyzed oxidation of styrene (Scheme 1b). Incubation of CYP152A1 (0.25 mg mL^–1^, 5.0 μM) with styrene (5 mM) and H_2_O_2_ (0.25 mM, 0.5 mM, or 1 mM) in the presence of heptanoic acid (10 mM) for 60 s generated styrene oxide and phenylacetaldehyde (Figure S5), as previously reported.^16^ As the initial concentration of H_2_O_2_ was increased, CYP152A1 produced more styrene oxide and phenylacetaldehyde (Table 2). Estimated product yields were approximately 14%, irrespective of the initial H_2_O_2_ concentration (Table 2). We confirmed that H_2_O_2_ was consumed by the catalase activity of CYP152A1 even in the reaction with styrene (Figure S4b). These results indicate that the amount of styrene oxide and phenylacetaldehyde produced by CYP152A1 also depends on the initial H_2_O_2_ concentration.

**Table 2:** Effect of H_2_O_2_ Concentration on CYP152A1-Catalyzed Synthesis of Styrene Oxide and Phenylacetaldehyde

H_2_O_2_ (mM)	styrene oxide (μM)	phenylacetaldehyde (μM)	product yield (%)^a^
0.25	21.6 ± 2.6	13.2 ± 1.3	13.9 ± 1.6
0.5	46.3 ± 1.5	28.6 ± 1.5	15.0 ± 0.6
1.0	89.0 ± 4.5	53.0 ± 4.9	14.2 ± 0.9

a Product yield (%) based on H_2_O_2_ expressed as (styrene oxide produced [mol] + phenylacetaldehyde produced [mol])/(H_2_O_2_ added [mol]) × 100.

### SCG- and TLR-Driven CYP152A1-Catalyzed Synthesis of Russig’s Blue

**Figure 2:**
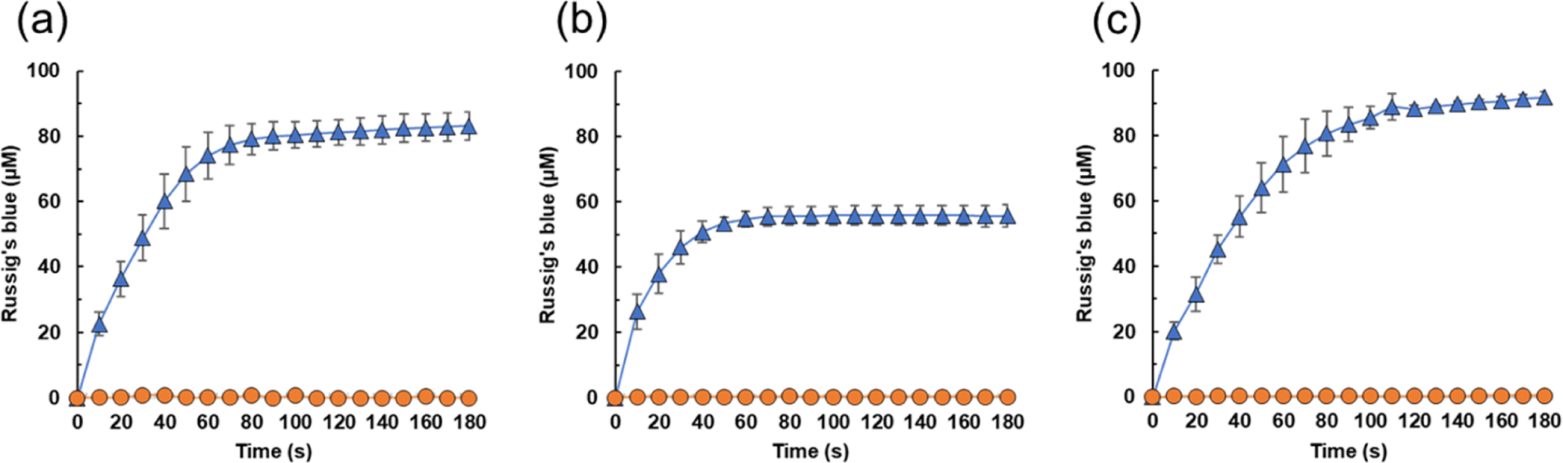
SCG- and TLR-driven CYP152A1-catalyzed synthesis of Russig’s blue. Enzyme and substrate solution (500 μL) containing CYP152A1 (0.5 mg mL^–1^, 10 μM), 4-methoxy-1-naphthol (2 mM), and heptanoic acid (20 mM) in sodium phosphate buffer was mixed with H_2_O_2_ solution (500 μL each) prepared by the incubation of pyrogallol (a), SCG (b), or TLR (c) in sodium phosphate buffer for 24 h. Reactions were carried out for 180 s in the absence (circles) or presence (triangles) of CYP152A1. Data are the average of three independent experiments, and error bars indicate the standard deviation from the mean.

We investigated the oxidation of 4-methoxy-1-naphthol to Russig’s blue using H_2_O_2_ generated from SCG and TLR as a model reaction. An enzyme and substrate solution (500 μL) containing CYP152A1 (0.5 mg mL^–1^, 10 μM), 4-methoxy-1-naphthol (2 mM), and heptanoic acid (20 mM) in sodium phosphate buffer was mixed with H_2_O_2_ solutions (500 μL each) prepared by incubating SCG or TLR in sodium phosphate buffer for 24 h, as illustrated in Figure 1. Both SCG-derived and TLR-derived H_2_O_2_ promoted the CYP152A1-catalyzed oxidation of 4-methoxy-1-naphthol, with 56.2 and 91.8 μM of Russig’s blue formed during a 180 s reaction in the mixtures containing SCG-derived H_2_O_2_ and TLR-derived H_2_O_2_, respectively (Figure 2b,c). It should be noted that in the absence of CYP152A1, no product formation was observed. The H_2_O_2_ solution prepared from pyrogallol also functioned as an oxidant (Figure 2a). The initial concentration of SCG-derived H_2_O_2_ was lower than that of pyrogallol-derived H_2_O_2_, resulting in a lower amount of Russig’s blue produced with SCG-derived H_2_O_2_ than with pyrogallol-derived H_2_O_2_ (Table 3). Almost the same amount of Russig’s blue was produced with TLR-derived H_2_O_2_ as with pyrogallol-derived H_2_O_2_ (Table 3). These results suggest that components in the SCG and TLR solutions do not inhibit the CYP152A1-catalyzed reaction. Estimated product yields based on H_2_O_2_ were 69.3 and 42.0% for SCG and TLR, respectively (Table 3). The yield for SCG was much higher than that for the reagent H_2_O_2_ (48%) (Table 1). Although we cannot fully explain this difference, the TOF data suggest that various components in SCG might accelerate the synthesis of Russig’s blue (Table 3). Nevertheless, these results clearly demonstrate that the abundant waste biomass sources SCG and TLR promote oxidation biocatalysis.

**Table 3:** SCG- and TLR-Driven CYP152A1-Catalyzed Synthesis of Russig’s Blue

electron donor	H_2_O_2_ (mM)^a^	Russig’s blue (μM)	product yield (%)^b^	TOF (min^–1^)^c^
Pyrogallol	0.48 ± 0.01	83.1 ± 4.4	34.4 ± 1.8	27.0 ± 4.2
SCGs	0.16 ± 0.01	56.2 ± 3.2	69.3 ± 3.9	31.5 ± 6.5
TLRs	0.44 ± 0.02	91.8 ± 1.7	42.0 ± 0.8	24.1 ± 3.2

a The H_2_O_2_ solution was diluted twofold before mixing with the enzyme and substrate solution to enable real-time spectrophotometric monitoring of the formation of Russig’s blue over the range in which concentration is proportional to absorbance.

b Product yield (%) based on H_2_O_2_ expressed as (2 × Russig’s blue produced [mol])/(H_2_O_2_ added [mol]) × 100.

c TOF (min^–1^) was estimated for the first 10 s of the reaction.

### SCG- and TLR-Driven CYP152A1-Catalyzed Synthesis of Styrene Oxide and Phenylacetaldehyde

**Figure 3:**
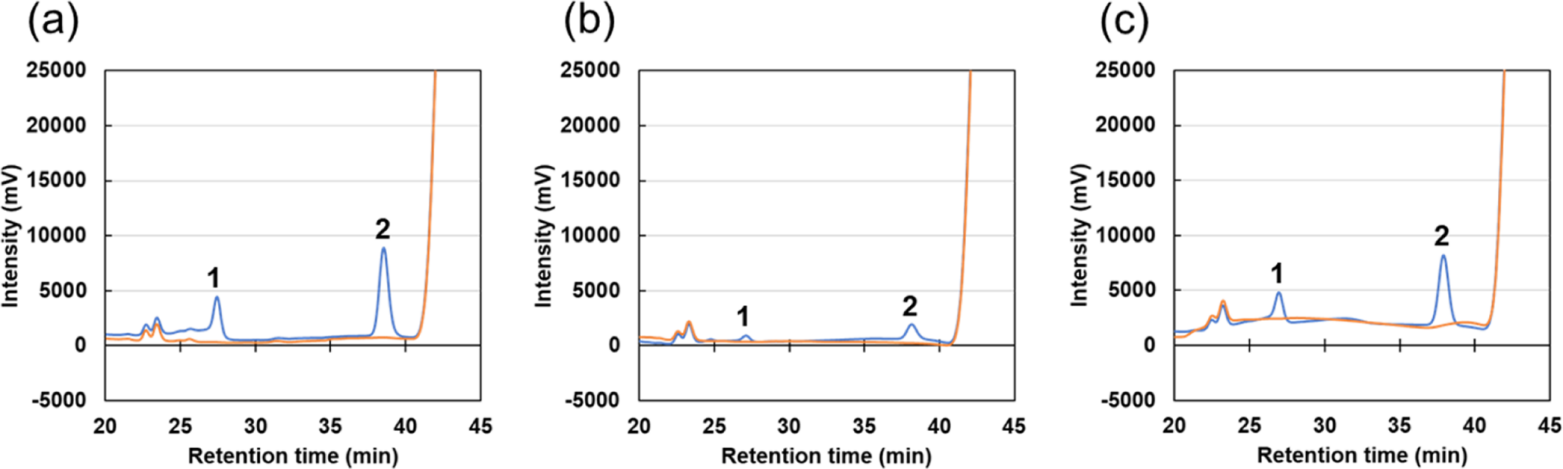
SCG- and TLR-driven CYP152A1-catalyzed synthesis of styrene oxide and phenylacetaldehyde. Enzyme and substrate solution (500 μL) containing CYP152A1 (0.5 mg mL^–1^, 10 μM), styrene (2 mM), and heptanoic acid (20 mM) in sodium phosphate buffer was mixed with H_2_O_2_ solution (500 μL each) prepared by the incubation of pyrogallol (a), SCG (b), or TLR (c) in sodium phosphate buffer for 24 h. Reactions were carried out for 60 s in the presence (blue lines) or absence (red lines) of CYP152A1. Peaks 1 (at 27.3 min) and 2 (at 38.4 min) in HPLC analysis correspond to phenylacetaldehyde and styrene oxide, respectively.

We also investigated the oxidation of styrene to styrene oxide and phenylacetaldehyde. CYP152A1 was incubated with styrene, heptanoic acid, and H_2_O_2_ prepared from SCG and TLR. Both SCG-derived and TLR-derived H_2_O_2_ promoted the CYP152A1-catalyzed oxidation of styrene (Figure 3). SCG promoted the synthesis of 13.9 μM styrene oxide and 9.8 μM phenylacetaldehyde during a 60 s reaction (Table 4). The TLR solution contained a higher amount of H_2_O_2_ compared with the SCG solution and therefore promoted the synthesis of more styrene oxide and phenylacetaldehyde (63.6 and 44.1 μM, respectively) (Table 4). Estimated product yields were 7.3 and 12.3% for SCG and TLR, respectively (Table 4). These values were almost the same as (or slightly lower than) those for the reagent H_2_O_2_ (14%) (Table 2). These results again demonstrate that SCG and TLR solutions function well as sources of H_2_O_2_ to drive the CYP152A1-catalyzed synthesis of styrene oxide and phenylacetaldehyde.

**Table 4:** SCG- and TLR-Driven CYP152A1-Catalyzed Synthesis of Styrene Oxide and Phenylacetaldehyde

electron donor	H_2_O_2_ (mM)	styrene oxide (μM)	phenylacetaldehyde (μM)	product yield (%)^a^
Pyrogallol	0.97 ± 0.02	73.6 ± 2.7	58.1 ± 5.6	13.6 ± 0.8
SCGs	0.32 ± 0.01	13.9 ± 1.6	9.8 ± 0.8	7.3 ± 0.6
TLRs	0.87 ± 0.04	63.6 ± 2.0	44.1 ± 1.9	12.3 ± 0.2

a Product yield (%) based on H_2_O_2_ expressed as (styrene oxide produced [mol] + phenylacetaldehyde produced [mol])/(H_2_O_2_ added [mol]) × 100.

**Figure 4:**
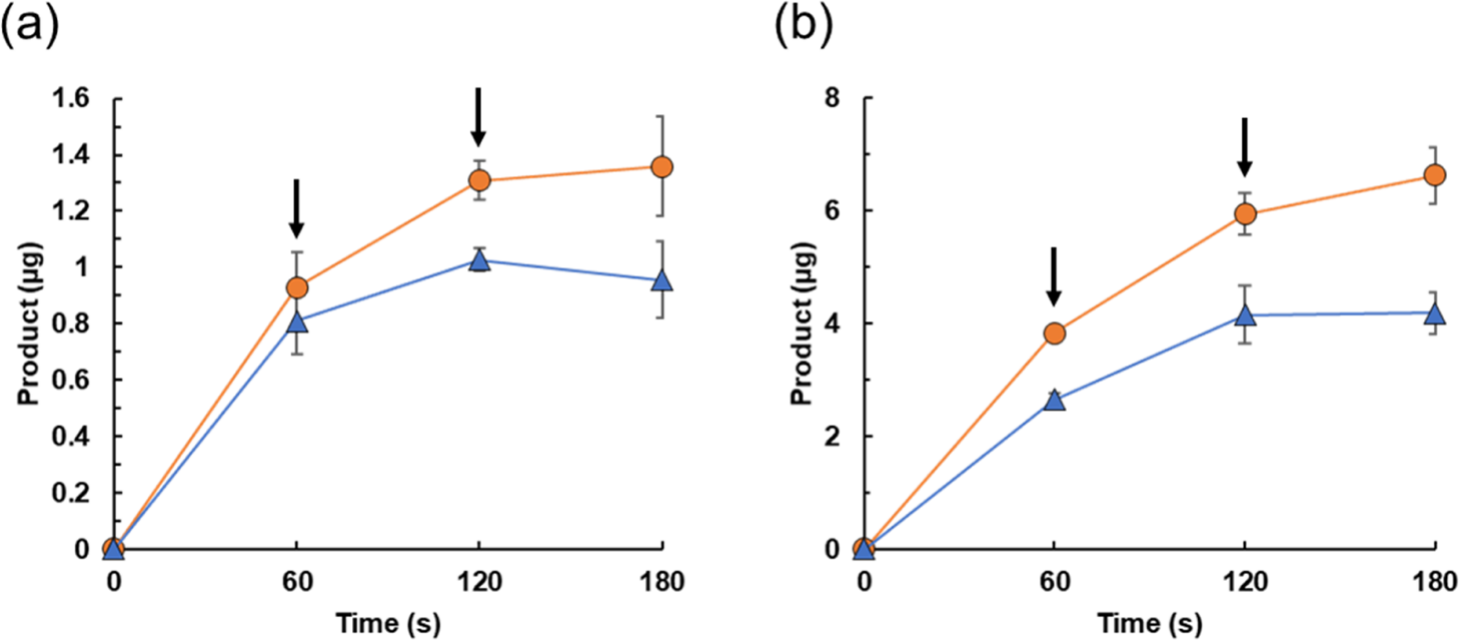
CYP152A1-catalyzed synthesis of styrene oxide and phenylacetaldehyde with repeated addition of H_2_O_2_ solution. Enzyme and substrate solution (250 μL) containing CYP152A1 (0.5 mg mL^–1^, 10 μM), styrene (10 mM), and heptanoic acid (20 mM) in sodium phosphate buffer was mixed with H_2_O_2_ solution (250 μL each) prepared by the incubation of SCG (a) or TLR (b) in sodium phosphate buffer for 24 h. After incubation for 60 and 120 s, H_2_O_2_ solution (250 μL each) was repeatedly added to the reaction mixture (indicated by arrows). Styrene oxide (circles) and phenylacetaldehyde (triangles) were determined by HPLC. Data are the average of three independent experiments, and error bars indicate the standard deviation from the mean.

We further attempted to produce styrene oxide and phenylacetaldehyde via repeated addition of SCG-derived H_2_O_2_ to the reaction mixture, as H_2_O_2_ in the mixture was rapidly consumed by the catalase activity of CYP152A1 (Figure S4b). In the presence of SCG solution, CYP152A1 produced 0.93 μg (7.7 nmol) of styrene oxide and 0.81 μg (6.7 nmol) of phenylacetaldehyde in the microtube-scale analysis during a 60 s reaction (Figure 4a and Table S1). After the reaction, SCG solution was again added to the mixture. CYP152A1 retained its activity, enabling the production of 1.31 μg (10.9 nmol) of styrene oxide and 1.03 μg (8.6 nmol) of phenylacetaldehyde. In subsequent reactions, with addition of SCG solution, product amounts did not increase, probably due to inactivation of the enzyme. Using a similar technique, repeated addition of TLR to the reaction mixture resulted in the production of 6.63 μg (55.2 nmol) and 4.18 μg (34.8 nmol) of styrene oxide and phenylacetaldehyde, respectively (Figure 4b and Table S2).

## Conclusions

H_2_O_2_ is an important and versatile industrial compound useful in numerous applications. H_2_O_2_ is produced industrially primarily via the anthraquinone process, although extensive research has focused on more environmentally benign methods.^17−21^ In the present study, we found that H_2_O_2_ could be effectively produced from the waste biomass sources SCG and TLR by reacting them with O_2_ in sodium phosphate buffer. These results indicate new applications of SCG and TLR as sources of H_2_O_2_. A more detailed examination of the reaction conditions should enable further enhancement of H_2_O_2_ productivity. The method in this work is inexpensive due to the lack of need for catalysts, whereas combining SCG/TLR with photocatalysts or electrocatalysts, which have been recently reported,^40−42^ might provide an effective means for high-efficiency H_2_O_2_ production. We also examined the synthesis of valuable chemicals by supplying SCG- and TLR-derived H_2_O_2_ for the oxidation of biocatalyst CYP152A1. We demonstrated that both SCG-derived and TLR-derived H_2_O_2_ promote CYP152A1-catalyzed synthesis of Russig’s blue and styrene oxide. Furthermore, repeated addition of SCG and TLR to the reaction mixture enhanced the synthesis of styrene oxide and phenylacetaldehyde. CYP152A1 is an important prototypical bacterial P450 peroxygenase, but its functional stability is reportedly relatively low.^43^ Further investigations will therefore focus on immobilization of CYP152A1 as a means of synthesizing higher amounts of styrene oxide and other oxidized chemicals using SCG and TLR.^43,44^ In addition, new peroxygenases with excellent catalytic properties, such as CYP119, unspecific peroxygenases, and their variants, have been recently reported.^17,18,45−48^ The sustainable approach presented here should be readily applicable to these peroxygenases for the synthesis of a variety of valuable chemicals.

## Experimental Section

### H_2_O_2_ Production from Waste Biomass

Coffee and tea were prepared by extraction of coffee grounds (Unimat Life Corp., Tokyo, Japan) and tea leaves (ITO EN, Tokyo, Japan) (0.2 g of each) using distilled-deionized water (10 mL) heated at 90 °C for 10 min, with subsequent filtration. The resulting SCG and TLR were dried on the filter, and 0.02 g of each was added to water (250 μL). A solution of pyrogallol as a model polyphenol compound (5 mM) was also prepared. Each solution of coffee, tea, SCG, TLR, and pyrogallol (250 μL) was added to a microtube containing water or sodium phosphate buffer (100 mM [pH 7.4]; 250 μL). These solutions (500 μL total) were incubated at 30 °C with shaking in the dark for 48 h. After incubation, H_2_O_2_ generation was immediately measured using a FOX assay, as reported previously.^34^

### Expression of the CYP152A1 Gene in *E. coli*

The gene encoding CYP152A1 of *B. subtilis* (GenBank accession number, CAB12004) was cloned into pET-28a(+) (Novagen, Darmstadt, Germany) to obtain a gene product with an N-terminal His-tag. The CYP152A1 gene was amplified from the pET-21a(+) vector carrying the CYP152A1 gene^49,50^ by PCR using the oligonucleotide primers CGC GGA TCC GAT GAA TGA GC A GAT TCC ACA (*Bam*HI restriction site underlined) and ATA AGA ATG CGG CCG CTT AAC TTT TTC GTC TGA TT (*Not*I restriction site underlined) and then inserted into pET-28a(+) via *Bam*HI/*Not*I sites. The resulting plasmid was introduced into *E. coli* Rosetta 2(DE3) cells (Novagen). Transformed *E. coli* cells were cultivated at 30 °C in LB medium containing (per liter) Bacto Tryptone (10 g), Bacto yeast extract (5 g), and NaCl (10 g) (pH 7.0) and supplemented with kanamycin (50 μg mL^–1^) and chloramphenicol (30 μg mL^–1^). After cultivation for 6 h (OD_600_ = 0.8–1.0), isopropyl-β-d-thiogalactopyranoside (1 mM), 5-aminolevulinic acid (0.5 mM), and FeSO_4_ (0.5 mM) were added to the medium, and cultivation was continued for an additional 15 h at 25 °C. Cells were harvested by centrifugation and washed with potassium phosphate buffer (200 mM [pH 7.5]) containing glycerol (10% [v/v]) and used for protein expression and purification.

### Purification of CYP152A1

CYP152A1 with an N-terminal His-tag was purified from the soluble fraction of transformed *E. coli* cells using a HisTrap HP column (Cytiva, Marlborough, MA, USA) according to the instruction manual. The soluble fraction was applied to a HisTrap HP 1 mL column equilibrated with sodium phosphate (20 mM [pH 7.4]) containing NaCl (500 mM) and imidazole (20 mM). The column was then washed with 10 column volumes of the same buffer. The bound His-tagged protein was eluted with sodium phosphate (20 mM [pH 7.4]) containing NaCl (500 mM) and imidazole (500 mM). Individual fractions were analyzed by SDS-PAGE, and those containing CYP152A1 were combined and desalted using a HiTrap desalting column (Cytiva) equilibrated with sodium phosphate buffer (100 mM [pH 7.4]) containing glycerol (10% [v/v]) to remove imidazole. The protein concentration was determined using a Coomassie protein assay kit (Pierce, Rockford, IL, USA) with bovine serum albumin as the standard.^51^

### CYP152A1-Catalyzed Oxidation

In the oxidation reaction of 4-methoxy-1-naphthol with CYP152A1, the reaction mixture (1 mL) contained purified CYP152A1 (49.7 kDa, 0.25 mg mL^–1^, 5.0 μM), 4-methoxy-1-naphthol (1 mM, Tokyo Kasei, Tokyo, Japan), dimethylsulfoxide (1% [v/v]), heptanoic acid (10 mM) as a decoy molecule, and H_2_O_2_ (0.1 mM, 0.25 mM, or 0.5 mM) in sodium phosphate buffer (50 mM [pH 7.4]). The reactions were carried out in cuvettes for 120 s, and formation of Russig’s blue was monitored spectrophotometrically at a wavelength of 610 nm. The concentration of Russig’s blue was calculated using an extinction coefficient of 1.45 × 10^4^ M^–1^ cm^–1^ at 610 nm.^52^ Product yield (%) based on H_2_O_2_ was expressed as (2 × Russig’s blue produced [mol])/(H_2_O_2_ added [mol]) × 100. The factor of “2” in this equation was required because two molecules of H_2_O_2_ are consumed to produce one molecule of Russig’s blue (Scheme 1a). The TOF (min^–1^) was estimated for the first 10 s of the reaction.

In the oxidation reaction of styrene with CYP152A1, the reaction mixture (500 μL) contained purified CYP152A1 (0.25 mg mL^–1^, 5.0 μM), styrene (5 mM, Fujifilm Wako Chemicals, Osaka, Japan), dimethylsulfoxide (1% [v/v]), heptanoic acid (10 mM), and H_2_O_2_ (0.25 mM, 0.5 mM, or 1 mM) in sodium phosphate buffer (50 mM [pH 7.4]). Reactions were carried out in microtubes for 60 s. Generation of styrene oxide and phenylacetaldehyde was immediately assessed by high-performance liquid chromatography (HPLC), as described below. Product yield (%) based on H_2_O_2_ was expressed as (styrene oxide produced [mol] + phenylacetaldehyde produced [mol])/(H_2_O_2_ added [mol]) × 100 (Scheme 1b).

### SCG- and TLR-Driven CYP152A1-Catalyzed Oxidation

In the oxidation reaction of 4-methoxy-1-naphthol with CYP152A1, the enzyme and substrate solution contained purified CYP152A1 (0.5 mg mL^–1^, 10 μM), 4-methoxy-1-naphthol (2 mM), dimethylsulfoxide (2% [v/v]), and heptanoic acid (20 mM) in sodium phosphate buffer (100 mM [pH 7.4]). H_2_O_2_ was generated by incubation of SCG or TLR in sodium phosphate buffer for 24 h, as described above, with subsequent filtration. The resulting H_2_O_2_ solution was diluted twofold before mixing with the enzyme and substrate solution to enable real-time spectrophotometric monitoring of the formation of Russig’s blue over the range in which concentration is proportional to absorbance. The enzyme and substrate solution (500 μL) was mixed with the twofold-diluted H_2_O_2_ solution (500 μL) in a cuvette and incubated for 180 s with spectrophotometric monitoring of Russig’s blue formation at a wavelength of 610 nm.

In the oxidation reaction of styrene with CYP152A1, the enzyme and substrate solution contained purified CYP152A1 (0.5 mg mL^–1^, 10 μM), styrene (10 mM), dimethylsulfoxide (2% [v/v]), and heptanoic acid (20 mM) in sodium phosphate buffer (100 mM [pH 7.4]). H_2_O_2_ was generated by incubation of SCG or TLR in sodium phosphate buffer for 24 h, as described above, with subsequent filtration. The enzyme and substrate solution (250 μL) was then mixed with a H_2_O_2_ solution (250 μL) in a microtube and incubated for 60 s. The resulting H_2_O_2_ solution (250 μL) was repeatedly added to the mixture at 60 s intervals when required. Generation of styrene oxide and phenylacetaldehyde was immediately analyzed by HPLC, as described below.

### HPLC Analysis

Reaction products of styrene oxidation were analyzed by HPLC using an LC-20 system (Shimadzu, Kyoto, Japan) equipped with a COSMOSIL 5C18-PAQ packed column (4.6 × 250 mm, Nacalai Tesque, Kyoto, Japan).^53,54^ Ethyl acetate (volume identical to that of the reaction mixture) was added to the post-reaction mixture. The solution was then vigorously shaken and centrifuged, and the resulting supernatant (5 μL) was injected into the HPLC system. Mobile phases were water (A) and methanol (B). A gradient of mobile phase B was programmed as follows: a start ratio of 35%, held at 35% for 29 min, increased to 100% over 1 min, held at 100% for 10 min, decreased to 35% over 1 min, and held at 35% for 17 min. The flow rate was 0.5 mL min^–1^. Compounds were detected spectrophotometrically at a wavelength of 210 nm. The amounts of styrene oxide and phenylacetaldehyde generated were calculated from standard calibration curves prepared using commercially available compounds.

## Supporting Information Available

CYP152A1-catalyzed synthesis of styrene oxide and phenylacetaldehyde with repeated addition of SCG solution, CYP152A1-catalyzed synthesis of styrene oxide and phenylacetaldehyde with repeated addition of TLR solution, polyphenols in coffee and tea as pro-oxidants, SDS-PAGE analysis of purification of CYP152A1, effect of H_2_O_2_ concentrations on CYP152A1-catalyzed synthesis of Russig’s blue, H_2_O_2_ consumption in the CYP152A1-catalyzed reactions, and effect of H_2_O_2_ concentrations on CYP152A1-catalyzed synthesis of styrene oxide and phenylacetaldehyde (PDF)
