# Supplementary material for: Sustainable Approach for Peroxygenase-Catalyzed Oxidation Reactions Using Hydrogen Peroxide Generated from Spent Coffee Grounds and Tea Leaf Residues
Source: ACS Omega. 2022 Jun 1;7(23):20259–66. doi: 10.1021/acsomega.2c02186 (PMC9201881; doi:10.1021/acsomega.2c02186)
Supplement: Supplementary file 1 — ao2c02186_si_001.pdf [file ao2c02186_si_001.pdf]

Supporting Information

Sustainable Approach for Peroxygenase-Catalyzed Oxidation Reactions Using Hydrogen Peroxide Generated from Spent Coffee Grounds and Tea Leaf Residues

Hideaki Kawana, Toru Miwa, Yuki Honda, and Toshiki Furuya

Table S1. CYP152A1-catalyzed synthesis of styrene oxide and phenylacetaldehyde with repeated addition of SCG solution.

Reaction time	Addition	Total volume of reaction (μL)	Concentration of styrene oxide (μM)	Total styrene oxide produced (μg)	Concentration of phenylacetaldehyde (μM)	Total phenylacetaldehyde produced (μg)
0 s to 60 s	-	500	15.4	0.93	13.5	0.81
60 s to 120 s	250 μL of SCG solution	750	14.5	1.31	11.4	1.03
120 s to 180 s	250 μL of SCG solution	1000	11.3	1.36	7.9	0.95

Table S2. CYP152A1-catalyzed synthesis of styrene oxide and phenylacetaldehyde with repeated addition of TLR solution.

Reaction time	Addition	Total volume of reaction (μL)	Concentration of styrene oxide (μM)	Total styrene oxide produced (μg)	Concentration of phenylacetaldehyde (μM)	Total phenylacetaldehyde produced (μg)
0 s to 60 s	-	500	63.6	3.82	44.1	2.65
60 s to 120 s	250 μL of TLR solution	750	65.9	5.94	46.0	4.15
120 s to 180 s	250 μL of TLR solution	1000	55.2	6.63	34.8	4.18

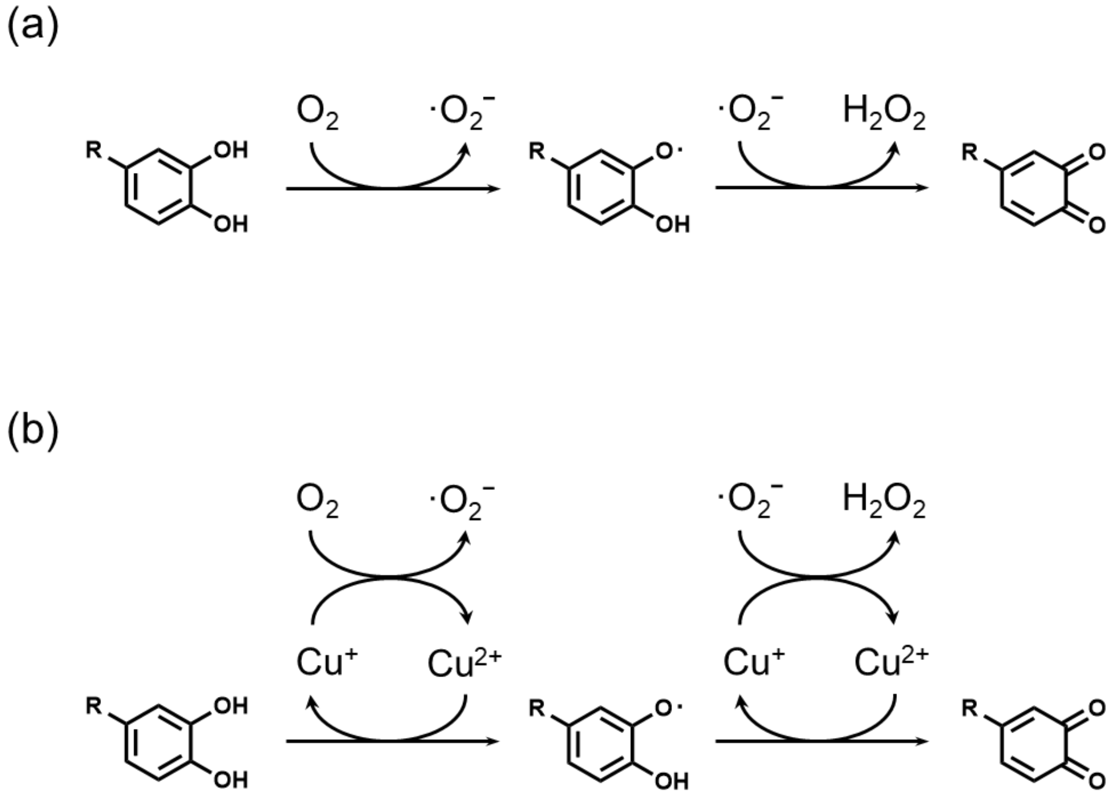

Figure S1. Polyphenols in coffee and tea as pro-oxidants. (a) They reduce O_2 to form the oxidant H_2O_2. (b) Traces of metal ions (e.g., copper and iron ions) in sodium phosphate buffer catalyze the reduction of O_2 by polyphenols to generate H_2O_2.

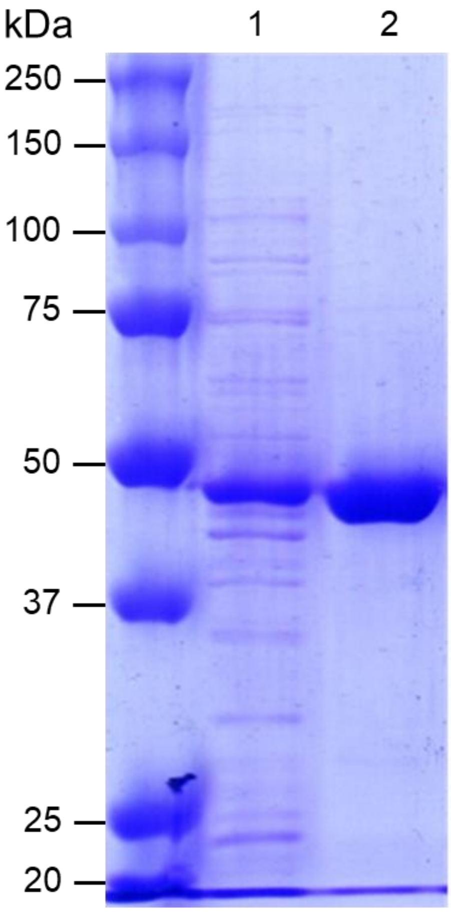

Figure S2. SDS-PAGE analysis of purification of CYP152A1. CYP152A1 with an N-terminal His-tag (49.7 kDa) was purified from the soluble fraction of transformed *E. coli* cells using a HisTrap HP column. 1, soluble-fraction sample; 2. purified CYP152A1 sample.

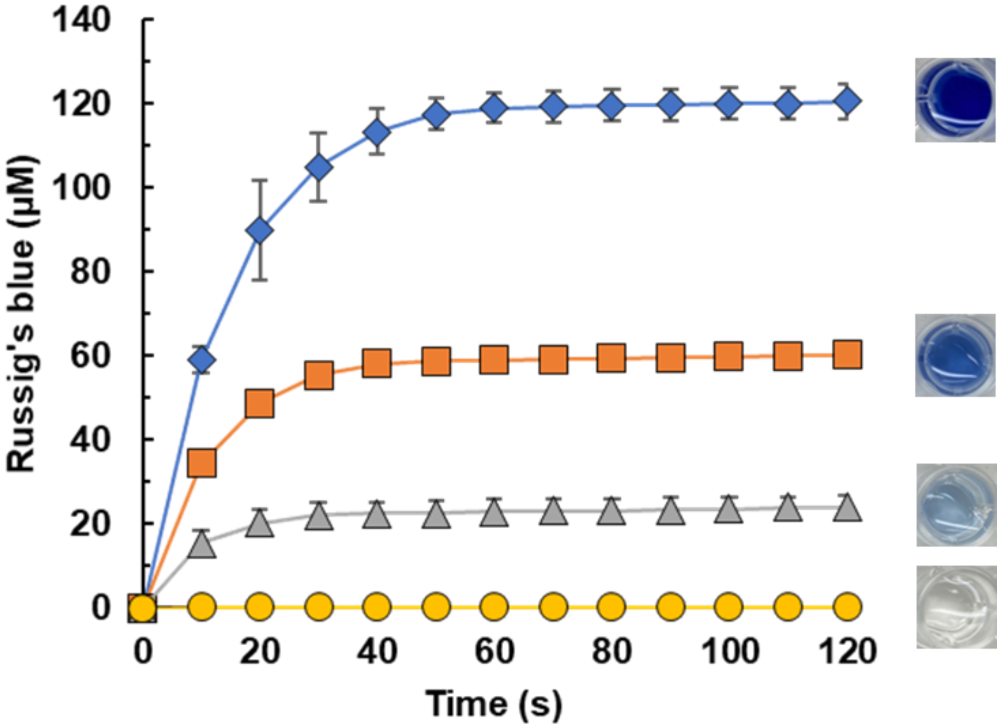

Figure S3. Effect of H_2O_2 concentration on CYP152A1-catalyzed synthesis of Russig's blue. CYP152A1 (0.25 mg mL^{-1}, 5.0 μM) was incubated with 4-methoxy-1-naphthol (1 mM) and H_2O_2 (0.1 mM, 0.25 mM, or 0.5 mM) in the presence of heptanoic acid (10 mM) for 120 s. The reaction mixtures turned blue due to the formation of Russig's blue. Triangles, 0.1 mM H_2O_2; squares, 0.25 mM H_2O_2; diamonds, 0.5 mM H_2O_2. Circles, 0.5 mM H_2O_2 in the absence of CYP152A1. Data are the average of three independent experiments, and error bars indicate the standard deviation from the mean.

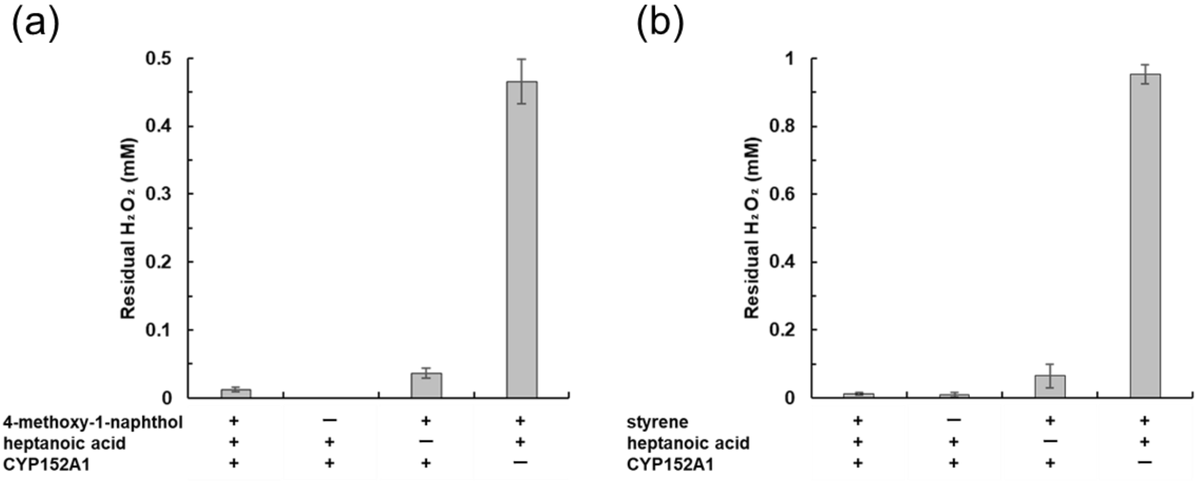

Figure S4. H_2O_2 consumption in the CYP152A1-catalyzed reactions. (a) CYP152A1 (0.25 mg mL^{-1}, 5.0 μM) was incubated with 4-methoxy-1-naphthol (1 mM) and H_2O_2 (0.5 mM) in the presence of heptanoic acid (10 mM) for 120 s under various conditions. (b) CYP152A1 (0.25 mg mL^{-1}, 5.0 μM) was incubated with styrene (5 mM) and H_2O_2 (1 mM) in the presence of heptanoic acid (10 mM) for 60 s under various conditions. Residual H_2O_2 after the incubation was measured by the FOX assay. Data are the average of three independent experiments, and error bars indicate the standard deviation from the mean.

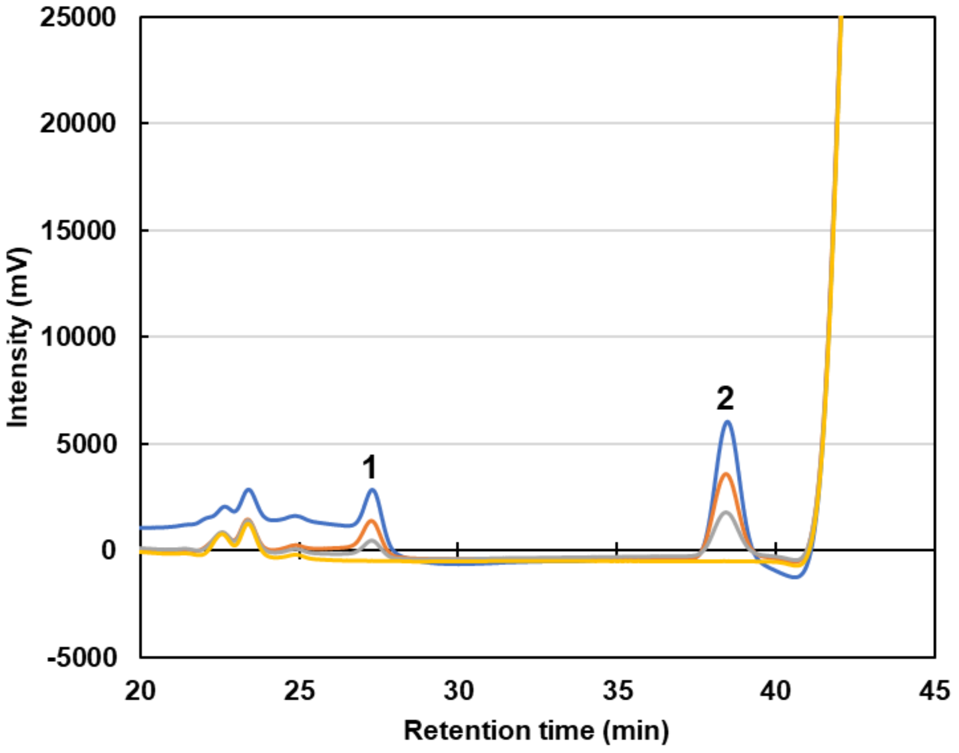

Figure S5. Effect of H_2O_2 concentration on CYP152A1-catalyzed synthesis of styrene oxide and phenylacetaldehyde. CYP152A1 (0.25 mg mL^{-1}, 5.0 μM) was incubated with styrene (5 mM) and H_2O_2 (0.25 mM, 0.5 mM, or 1 mM) in the presence of heptanoic acid (10 mM) for 60 s. Peaks 1 (at 27.3 min) and 2 (at 38.4 min) in HPLC analysis correspond to phenylacetaldehyde and styrene oxide, respectively. Gray line, 0.25 mM H_2O_2; red line, 0.5 mM H_2O_2; blue line, 1 mM H_2O_2. Yellow line, 1 mM H_2O_2 in the absence of CYP152A1.
